# Identification of arterial oxygen intermittency in oximetry data

**DOI:** 10.1038/s41598-022-20493-0

**Published:** 2022-09-26

**Authors:** Paulo P. Galuzio, Alhaji Cherif, Xia Tao, Ohnmar Thwin, Hanjie Zhang, Stephan Thijssen, Peter Kotanko

**Affiliations:** 1 Research Division, Renal Research Institute, New York, NY USA; 2 Icahn School of Medicine at Mount Sinai Health System, New York, NY USA

**Keywords:** Haemodialysis, Applied mathematics

## Abstract

In patients with kidney failure treated by hemodialysis, intradialytic arterial oxygen saturation (SaO_2_) time series present intermittent high-frequency high-amplitude oximetry patterns (IHHOP), which correlate with observed sleep-associated breathing disturbances. A new method for identifying such intermittent patterns is proposed. The method is based on the analysis of recurrence in the time series through the quantification of an optimal recurrence threshold (${{\varvec{\epsilon}}}_{\mathbf{o}\mathbf{p}\mathbf{t}}$). New time series for the value of ${{\varvec{\epsilon}}}_{\mathbf{o}\mathbf{p}\mathbf{t}}$ were constructed using a rolling window scheme, which allowed for real-time identification of the occurrence of IHHOPs. The results for the optimal recurrence threshold were confronted with standard metrics used in studies of obstructive sleep apnea, namely the oxygen desaturation index (ODI) and oxygen desaturation density (ODD). A high correlation between ${{\varvec{\epsilon}}}_{\mathbf{o}\mathbf{p}\mathbf{t}}$ and the ODD was observed. Using the value of the ODI as a surrogate to the apnea–hypopnea index (AHI), it was shown that the value of ${{\varvec{\epsilon}}}_{\mathbf{o}\mathbf{p}\mathbf{t}}$ distinguishes occurrences of sleep apnea with great accuracy. When subjected to binary classifiers, this newly proposed metric has great power for predicting the occurrences of sleep apnea-related events, as can be seen by the larger than 0.90 AUC observed in the ROC curve. Therefore, the optimal threshold ${{\varvec{\epsilon}}}_{\mathbf{o}\mathbf{p}\mathbf{t}}$ from recurrence analysis can be used as a metric to quantify the occurrence of abnormal behaviors in the arterial oxygen saturation time series.

## Introduction

Dynamic intermittency may refer to the irregular alternation of cyclic and/or recurrent behaviors^1^. Intermittency is seen in a broad range of systems undergoing shifts between distinct regimes. Identifying these patterns associated with pathological regime shifts provides a better understanding of the complex dynamics of these systems and is of utmost importance. In physiological systems, intermittent dynamics are sometimes seen as beneficial, especially when induced therapeutically, as observed in the treatment of osteoporosis^2^. However, in many contexts, intermittency signals dynamic transitions associated with pathophysiological shifts. For example, during sleep, an individual may experience sleep apnea, a condition where normal respiration is disrupted by episodes of apnea due to disturbed respiratory control or obstruction of upper airways, resulting in repetitive respiratory cessation. These repetitive dynamics may have characteristics of intermittent patterns seen in patients experiencing chronic obstructive pulmonary disease^3,4^, hypopnea, hypoxemia^5^, asthma, and stroke.

Arterial oxygen saturation (SaO_2_) is a key determinant of the amount of oxygen delivered to the tissues per unit of time. SaO_2_ is primarily determined by the structure and function of the respiratory system. Clinically, several invasive and non-invasive techniques are used to assess SaO_2_. Pulse oximetry has become ubiquitous in modern medicine, with applications ranging from monitoring patients in intensive care units^6,7^, during anesthesia, medical emergencies, and the diagnosis of respiratory illnesses. Recently, technologies have been introduced to continuously measure SaO_2_ in the extracorporeal blood circuit during hemodialysis. Such technologies have shown to be valuable for the identification of intradialytic hypoxemia^8–10^ and COVID-19 monitoring^11^, and other clinical outcomes^12^. When reviewing intradialytic SaO_2_ time series, we noticed a peculiar intermittent oscillatory SaO_2_ pattern. On occasion, we were able, through careful clinical observation, to correlate these intermittent high-frequency high-amplitude oximetry patterns (IHHOP) with sleep-associated breathing disturbances. This is relevant, as sleep-disordered breathing is frequent in dialysis patients^13^. The reported prevalence of sleep apnea is > 50% among dialysis patients^14–16^. Sleep apnea syndrome (SAS) is a condition that is associated with lower health-related quality of life^17^ and increased cardiovascular and mortality risk in kidney patients^18,19^. Our clinical observations led us to hypothesize that IHHOPs may be associated with patient outcomes. Evaluating this hypothesis will require the automated analysis of a large number of intradialytic SaO_2_ recordings and associate them with patient characteristics and outcomes. The goal of the clinical research presented here was to develop a methodology to automatically screen for IHHOPs in intradialytic SaO_2_ recordings. In addition, we explored the method’s utility to diagnose sleep apnea.

Currently, there are several biomarkers proposed for the analysis of Oxygen Saturation time series^7,20^, most of them fall into the categories of linear or statistical analysis, complexity measures, periodicity analysis, and desaturation analyses. For instance, several measures of central tendency, spread, or asymmetry^21,22^ have been used to characterize oxygen saturation levels in patients. Complexity measures, e.g. Lempel–Ziv complexity^23^ or different forms of entropy^24^ have also been employed. Characterizations of periodicity using phase-rectified signal averaging techniques^7,21,25^ or power spectrum density^26–31^ have been explored to detect the peak in the spectrum at a frequency associated with the period of desaturations^20^. Similarly, analysis of desaturations such as their depth, length, area, or time between successive desaturations^7,20,27,32–34^, and the Oxygen Desaturation Index (ODI)^35–37^ have been widely used. ODD and ODI are the most widely used proxied metrics for AHI. However, they may suffer from the lack of an agreed-upon definition^37^. When individually regressed against the AHI, most of these digital biomarkers have some degree of correlation with AHI. Furthermore, most of these quantifiers provide information on the occurrence of SAS after being used as features of complex machine learning algorithms, which are usually black boxes, harder to generalize, and subject to data drift and/or shift. The proposed method in this work presents discriminatory power and has fewer more interpretable parameters, and it does not depend on any specific definition of baseline or of any particular shape or form for the desaturations. These characteristics make the proposed method more robust and more generalizable. Further investigation is required to assess its predictive potential in different scenarios.

Even though there is no generally agreed-upon definition, SaO_2_ levels below 90% are usually considered an indication of hypoxemia^6,9,10,38,39^. However, analyzing SaO_2_ levels alone may be insufficient to correctly assess the patient’s oxygenation status. We hypothesize the possibility that dynamical variations on SaO_2_ time evolution, even when its average levels are consistently above the 90% threshold, may represent a risk even to otherwise healthy individuals.

## Methods

### Study population

We conducted a prospective observational pilot study in in-center chronic hemodialysis patients using arterio-venous fistula as vascular access. All study procedures were approved by the Western Institutional Review Board® (Protocol number: #20162001) and performed in accordance with the principles of the Declaration of Helsinki. Signed written informed consent forms were obtained from all participants for this study, and demographic data were collected using case report forms.

### Data collection and processing

Each subject was studied in two HD sessions. They were equipped with WatchPAT device (Itamar Medical Ltd, Israel), also known as Home Sleep Apnea Test (HSAT), which is FDA approved for obstructive sleep apnea testing that measures peripheral oxygen saturation and other parameters. SaO_2_ was measured in the extracorporeal dialysis circuit with a sampling frequency of 1 Hz, using a Crit-Line Monitor (Fresenius Medical Care, Waltham, MA) connected to a research laptop. Each subject was videotaped during hemodialysis to detect whether the subject slept or changed position during the treatment and video consent was taken prior to the study visit.

The SaO_2_ time series were subjected to the following pre-processing: (1) any value of SaO_2_ smaller than 75% was removed and the missing values were linearly interpolated; (2) following the procedure described by Schlotthauer^40^, the time series were subjected to a low-pass filter with a cutoff frequency of 0.25 Hz^41^. A sleep report was generated by the WatchPAT device, in which the time during dialysis that the patient was asleep is reported, with a classification of light and deep sleep.

#### Time series resampling

Crit-Line and other oximetry devices can collect SaO_2_ over a wide range of sampling frequencies. However, it is not unusual in clinical settings that the sampling frequency would be smaller than 1 Hz. Thus, any diagnostic technique needs to be robust at smaller sampling frequencies. For this reason, all the analyses presented in this work were calculated from the original time series at a 1 Hz sampling frequency, and at a resampled time series at 0.1 Hz. The resampling was performed by replacing every 10 s by its average. The resampling does not compromise the quality of the results since we are interested in a phenomenon that has a typical larger time scale^31^.

### Rolling analysis of time series

A rolling analysis of a time series is used to assess its stability and stationarity over time. Given a time series with $N$ points: $x(t)=\{x({t}_{1}),\hspace{0.17em}x({t}_{2}),\dots ,\hspace{0.17em}x({t}_{N})\}$, and a function $f(\overrightarrow{x})$ that extracts a single metric from a time series, we let ${\overrightarrow{x}}_{i\to j}$ to denote the section of the time series from ${t}_{i}$ to ${t}_{j}$. The rolling analysis of the time series with window size $W$ and window step $S$ generates a new time series for the metric, which is given by: $\{f({\overrightarrow{x}}_{1\to W}),\hspace{0.17em}f({\overrightarrow{x}}_{S\to S+W}),\dots ,\hspace{0.17em}f({\overrightarrow{x}}_{N-W\to N})\}$.

### Recurrence quantification analysis

Recurrence is the property of dynamical systems that quantitates the time the trajectory returns to a state arbitrarily close to its initial state, after a sufficiently long time. In time series analysis, recurrence can be characterized with the recurrence plot^42^, defined as:

$$ R_{{ij}}  = \Theta \left( {\epsilon  - \left\| {x\left( {t_{i} } \right) - x\left( {t_{j} } \right)} \right\|} \right), $$

where $\Theta (\cdot )$ is the Heaviside function, $\epsilon$ is the recurrence threshold, and any two points $x({t}_{i})$ and $x({t}_{j})$ are considered recurrent if the distance between them (measured by a given metric $\parallel \cdot \parallel$) is smaller than $\epsilon$.

The recurrence plot ${R}_{ij}$ is a binary matrix containing information on the recurrences in the time series and characterizes the behavior of the phase space trajectory with micro and macroscale features. To summarize these micro- and macroscale information, several quantifiers and metrics were proposed: recurrence rate, determinism, laminarity, trapping time, and others^43^. In this work, we will focus on the *Determinism* ($\mathrm{DET}$), which is a measure of how deterministic and predictable the analyzed time series is (e.g., periodic time series have determinism close to one, as random ones have determinism close to zero). DET is defined as:

$$\mathrm{DET}\left(\epsilon ;{\mathcalligra{l}}_{\mathrm{min}}\right)=\frac{\sum_{\mathcalligra{l}={\mathcalligra{l}}_{\mathrm{min}}}^{N}\mathcalligra{l}P\left(\mathcalligra{l},\epsilon \right)}{\sum_{\mathcalligra{l}=1}^{N}\mathcalligra{l}P\left(\mathcalligra{l},\epsilon \right)},$$

where $P\left(\mathcalligra{l}\right)$ is a histogram of diagonal lines of length $\mathcalligra{l}$ in the recurrence plot. A $\mathcalligra{l}$-long diagonal line in the recurrence plot is created when two distinct segments of the time series show similar behavior (within a tolerance of $\epsilon$) for at least $\mathcalligra{l}$ data points. Throughout this study the value ${\mathcalligra{l}}_{\mathrm{min}}=10$ was adopted, for time series with a sampling rate of 1 Hz, and ${\mathcalligra{l}}_{\mathrm{min}}=2$, for time series with a sampling rate of 0.1 Hz. The determination of the optimal value of ${\mathcalligra{l}}_{\mathrm{min}}$ is, to some extent, arbitrary, however, our results seem to suggest that reasonable values of ${\mathcalligra{l}}_{\mathrm{min}}$ should span an interval of around 10 s.

Under the assumption that the system dynamics show some degree of determinism, it is possible to define an optimal value for the recurrence threshold $\epsilon$^44^, such that:

$${\epsilon }_{\mathrm{opt}}\left({\mathcalligra{l}}_{\mathrm{min}}\right)=\underset{\epsilon }{\mathrm{arg}\hspace{0.17em}\mathrm{max}}\frac{d\hspace{0.17em}\mathrm{DET}\left(\epsilon ;{\mathcalligra{l}}_{\mathrm{min}}\right)}{d\epsilon }.$$

In this way, the value of ${\epsilon }_{\mathrm{opt}}$ brings information about the size of the system attractor, similarly to its variance, but also taking into consideration the characteristic behaviors of the dynamical system (e.g., periodic, and deterministic systems have smaller values of ${\epsilon }_{\mathrm{opt}}$ than stochastic ones). Although in previous studies, ${\epsilon }_{\mathrm{opt}}$ has been fixed to perform recurrence quantification analysis, we employ ${\epsilon }_{\mathrm{opt}}$ as a metric to characterize our time series. The calculation of ${\epsilon }_{\mathrm{opt}}$ assumes that the underlying system presents some degree of determinism, which is less strict than assuming periodicity of desaturations on time–frequency-based analysis^20^.

### Standard metrics for oximetry data

The diagnostics of obstructive sleep apnea–hypopnea syndrome (OSAHS) is based on the apnea–hypopnea index (AHI), which is the frequency of sleep-related breathing events per hour of sleep^45^. According to the American Sleep Disorders Association (ASDS), it can be classified as^33^ mild (5–15 events/h), moderate (15–30 events/h), and severe (> 30 events/h).

The ASDS guidelines dictate that the diagnosis should not be based solely on breathing events, but also consider other clinical factors. However, identifying the breathing obstruction events is still a major component of diagnosing OSAHS. The gold standard diagnosis tool is polysomnography (PSG), an expensive, time-consuming, and not always available technique. Therefore, many different works^34,36,46–48^ have proposed the use of oximetry data as a surrogate, for its low cost and accessibility. In this section, we briefly describe the most common metric developed to assess the occurrence of OSAHS from oximetry data, the oxygen desaturation index (ODI).

### Oxygen desaturation index (ODI) and oxygen desaturation density (ODD)

The most common use of oximetry involves the identification of oxygen desaturations, which is done by searching for drops on the oxygen saturation time series from a pre-established baseline. There are diverse ways of determining the baseline proposed in the literature, the most common being the average of oxygen saturation values for a short time (~ 3 min) at the beginning of the experiment.

In this work, we used the following procedure for identifying oxygen desaturation^20,40,46^.

- The baseline for each oxygen saturation time series is determined as the average of the first 3 min.
- A desaturation should drop at least N% from the baseline.
- The desaturation should remain below N% from baseline for at least 10 s, not exceeding 60 s.
- Each desaturation time stamp is at the instant the oxygen saturation first falls below N% from baseline.
- Usual values for N are 3% or 4%.

One of the issues using the above method in identifying breathing obstructions and diagnosing OSAHS is primarily due to its dependence on a baseline. This is particularly true for patients with other complications, such as patients undergoing dialysis. In which case, the values of oxygen saturation might be already low at the beginning of a dialysis section^8^, potentially compromising the reliability of the result^37^. Also, the ODI was designed specifically in the context of identifying sleep apnea patterns from oximetry data. However, it is not known how generalizable its results could be to the identification of other conditions.

Once the desaturations were identified, it is possible to calculate the ODI, which has been used as a surrogate for the AHI^36^. The ODIN represents the number of desaturations at least N% deep (say 3% or 4%) below baseline per hour of sleep. It is also possible to calculate an oxygen desaturations density (ODD): at any given point $t$, ODD is given by the count of how many desaturations were identified at the time series in the previous 10 min. In this way, it is possible to accompany the evolution of the OSAHS in time.

The main advantages of the proposed method compared to the ODD and ODI are: (1) the identification of oxygen desaturations is dependent on the definition of a baseline, which in most practical situations may be particularly difficult to determine, even more so for patients with comorbidities that can affect their Oxygen Saturation (as is the case of dialysis patients)^11,19^; (2) the ODI definition varies between authors, and they are usually tailored for the specific application of identifying sleep apnea events, by looking at a very specific shape in the oxygen saturation time series^37^; (3) since the definition of desaturations is so specific, deviations from most common scenarios can jeopardize its predictive ability, situations like dialysis patients or patients in high altitude already present reduced average of oxygen saturation, which can impact the ODI through the baseline calculation^19,49^; (4) some of the ODI definitions specify the desaturation minimal duration (usually 10 s), making them more difficult to identify in time series with low sampling frequency (e.g., 0.1 Hz)^20,40,46^. The proposed method is more robust against these shortcomings for the ODI: it does not depend on any baseline definition; it is also insensitive to the averages of the Oxygen Saturation; its definition is more precise, and it is robust against the sampling frequency of the input time series, and the only free parameter (${\mathcalligra{l}}_{\mathrm{min}}$) is directly interpretable in terms of the timescale of the problem.

## Results and discussion

### Patient population

A total of 16 subjects from a New York City dialysis clinic were studied. The mean age was 55 ± 10 years old; 63% were male and 69% were African American. Mean height 174.8 ± 9.4 cm, pre-dialysis weight 80.9 ± 22.5 kg, BMI 26.4 ± 6.9, HD vintage 5.7 ± 3.7 years. Mean SO_2_ was 94.3 ± 2.1%. As each subject was studied in two HD sessions, we analyzed 32 time series, with an average duration of 3.0 ± 0.5 h. Table 1 has a summary of dialysis treatment parameters, the classification of SAS in the table was made separately for each treatment.

**Table 1:** Summary of dialysis treatments parameters (mean ± SD).

Treatment parameters	All visits	Classification by SAS intensity				
		Healthy	Mild	Moderate	Severe	No sleep
Pre-HD weight (kg)	81.7 ± 23.3	72 ± 20.4	84.7 ± 22.4	76.4 ± 0	99.6 ± 27.3	83 ± 21.1
Post-HD weight (kg)	79.5 ± 23.1	69.8 ± 20.2	82.7 ± 22.4	73.9 ± 0	97.1 ± 26.3	80.8 ± 20.6
IDWG (kg)	2.2 ± 1	2.2 ± 0.9	2 ± 1.3	2.7 ± 0	3.1 ± 1	2.1 ± 0.9
Ultrafiltration volume (l)	2.2 ± 0.9	2.2 ± 1.2	2.1 ± 0.7	2.5 ± 0	2.5 ± 1	2.2 ± 1
Pre-HD SBP (mmHg)	148.9 ± 17.2	149.7 ± 20.5	149.4 ± 13.6	159 ± 0	134.7 ± 10.5	151.5 ± 16.2
Post-HD SBP (mmHg)	123.2 ± 14.6	124.1 ± 16.2	126.6 ± 17.3	106 ± 0	108.3 ± 11.6	124.9 ± 15.4
Treatment duration (min)	218.8 ± 32.9	214.6 ± 26.4	230.6 ± 34.1	211 ± 0	238.3 ± 41.9	210 ± 37.4
Sleep duration (min)	56 ± 59	81 ± 67	64 ± 31	32 ± 0	81 ± 48	N/A
eKT/V	1.6 ± 0.2	1.7 ± 0.3	1.7 ± 0.2	1.6 ± 0	1.6 ± 0	1.6 ± 0.2
Number of visits	32	13	7	1	3	8

### Identification of IHHOP

**Figure 1:**
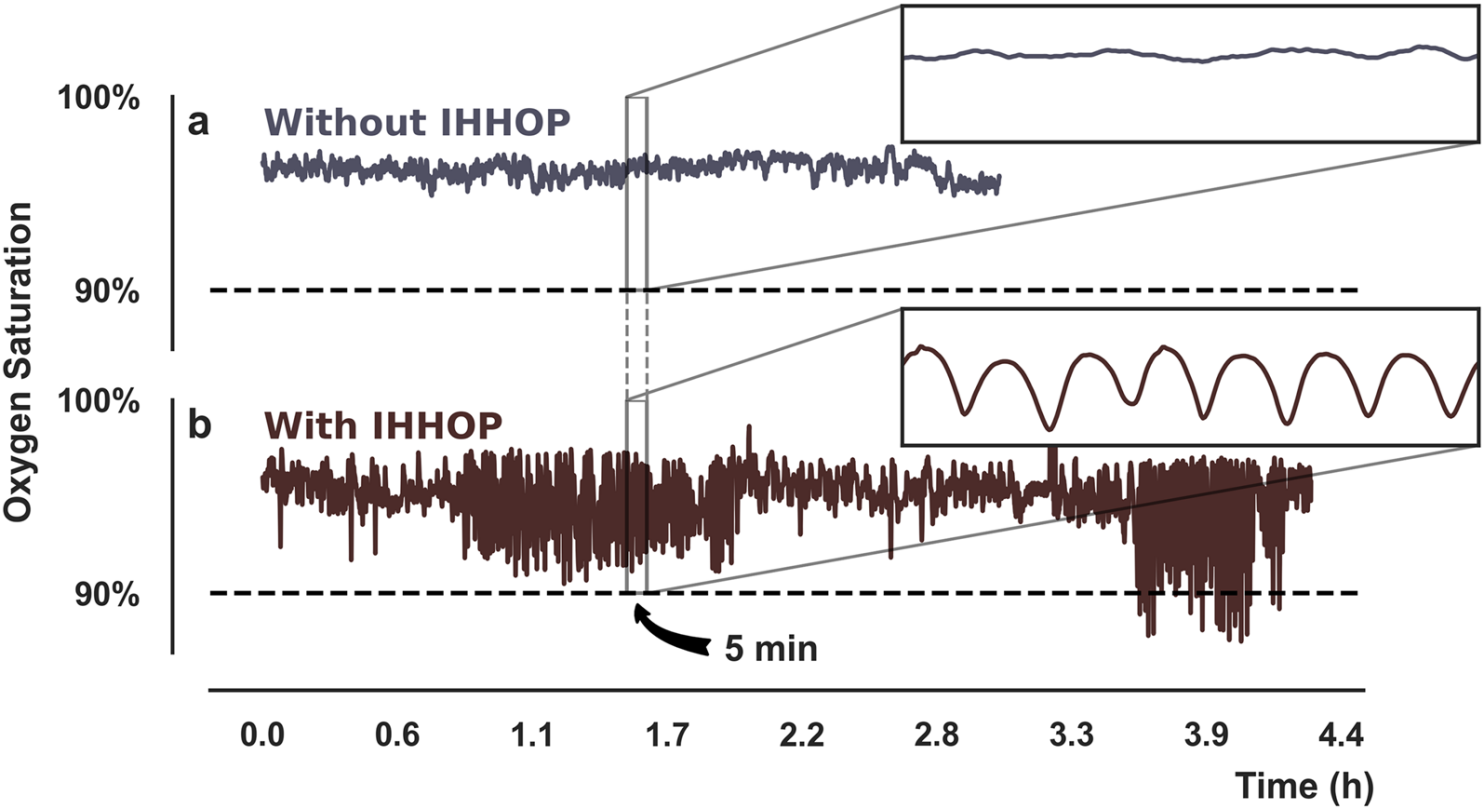
Illustrative arterial oxygen saturation time series from patients without intradialytic high-frequency and high-amplitude oximetry patterns (IHHOP) (**a**), and with IHHOP (**b**). The insets show 5 min segments of both time series. The dashed line marks the 90% oxygen saturation level, below which the patient is hypoxemic.

Figure 1 shows two illustrative examples of Oxygen Saturation using oximetry time series extracted from dialysis patients. In Fig. 1a, we observe a time series from a patient without the presence of IHHOP. Similarly, Fig. 1b shows an example of time series for a patient experiencing several episodes of IHHOP. The insets in Fig. 1 zoom into 5 min sections of the time series where the time series exhibiting IHHOP can be easily distinguishable from time series without IHHOP. An important aspect to notice is that some of the fluctuations do not fall below the 90% threshold, which is needed in order to be identified as a hypoxemic event under some clinical definition.

Generically, IHHOP differs from Intermittent Hypoxia^50–52^ in the sense that it is not sensitive to the absolute value of the oxygen saturation but only to its amplitude of variation and dynamical properties. IHHOP can only be characterized as intermittent Hypoxia by the occurrence of IHHOP with SaO_2_ below 90% as shown in some sections of Fig. 1b. Similarly, the occurrence of IHHOP is not in any way tied to the occurrence of hypoxemia, as can be seen in the early portion of the example shown in Fig. 1b. However, the systemic tissue oxygenation levels may be sensitive to the presence of IHHOP, and its identification is the first step towards understanding its pathophysiologic consequences and alleviating its potential adverse effects.

Many studies have looked into oximetry data in the context of sleep apnea^29,35,47^, where the main focus is on the identification of oxygen desaturations and their correlation to the Apnea–Hypopnea Index (AHI)^46,53^. Even though there is extensive literature investigating the relationship between Oxygen saturation and sleep apnea (and the patterns that arise in SaO_2_ time series), there is little or no general means of identifying the patterns investigated in this work beyond the scope of sleep studies.

**Figure 2:**
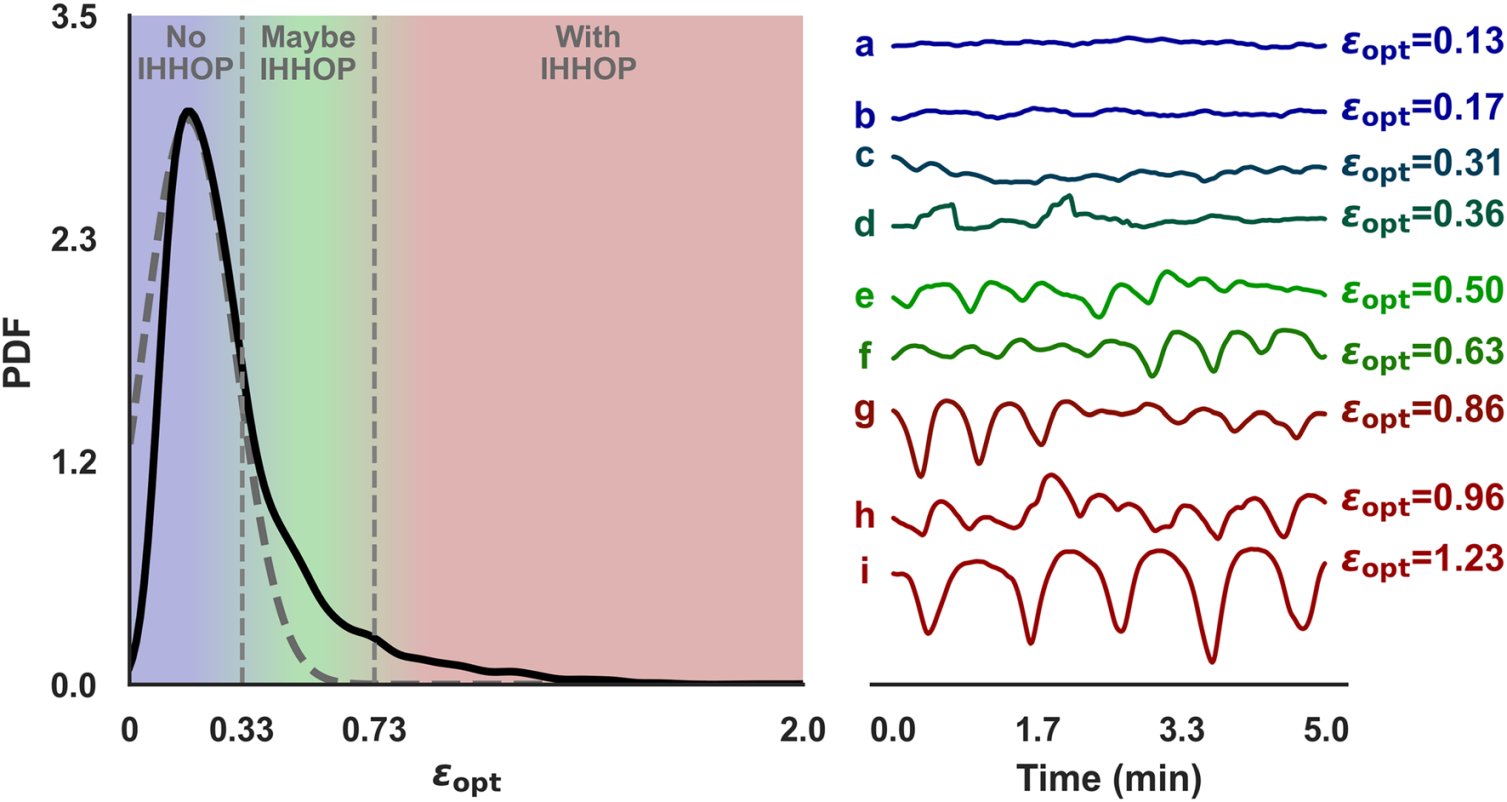
Distribution of values of ${\epsilon }_{opt}$ calculated from 5 min segments of SaO_2_ with 1.0 Hz sampling frequency, with a rolling window step size of 1 min, the vertically defined regions mark the clustering of the segments in three categories: no IHHOP (light blue), maybe IHHOP (light green) and with IHHOP (light red). On the right side, there are illustrative oxygen saturation 5-min segments for different values of ${\epsilon }_{opt}$. The y-axis is omitted to emphasize relative differences between the segments, all have the same scale for oxygen saturation values.

Given a segment of a SaO_2_ time series, the determination of whether it presents IHHOP depends on the comparison of its calculated value of ${\epsilon }_{\mathrm{opt}}$ against pre-established thresholds. To determine the values of such thresholds with the least amount of arbitrariness, it is important to understand how ${\epsilon }_{\mathrm{opt}}$ values are distributed for typical oxygen saturation time series. Figure 2 shows the probability distribution function (PDF) for the values of ${\epsilon }_{\mathrm{opt}}$ calculated from 5-min segments of SaO_2_ with 1.0 Hz sampling frequency, with a rolling window step size of 1 min, i.e., each 5 min segment overlaps 4 min of the next segment. The distribution shows a heavy tail, i.e., larger than normal probability of finding SaO_2_ segments with larger values of ${\epsilon }_{\mathrm{opt}}$. These higher probabilities for high values of ${\epsilon }_{\mathrm{opt}}$ are due to the presence of IHHOP in the respective time series segments. Heavy-tailed distributions are characteristic of intermittent dynamics and rare events^54,55^. This distribution can be naturally separated into at least two regions: (1) the region around its maximum point, where the distribution is similar to normal, and therefore values of ${\epsilon }_{\mathrm{opt}}$ in this region represent the normal/more frequent behavior of SaO_2_ dynamics; (2) the region of the heavy tail, which comprehends values of ${\epsilon }_{\mathrm{opt}}$ that characterize the intermittent dynamics.

Since a clear boundary for these two distinct dynamical behaviors cannot be defined, the full range of ${\epsilon }_{\mathrm{opt}}$ was separated into three regions: “No IHHOP” corresponds to ${\epsilon }_{\mathrm{opt}}\lesssim 0.33$; “Maybe IHHOP” $0.33\lesssim {\epsilon }_{\mathrm{opt}}\lesssim 0.73$; and “With IHHOP” $0.73\lesssim {\epsilon }_{\mathrm{opt}}$. The boundaries for these three classes were defined using Jenks natural breaks optimization^56,57^, however, their value should not be taken rigidly as they might change for different time series segments, sampling frequency, and value of ${\mathcalligra{l}}_{\mathrm{min}}$. An important aspect of these thresholds is that they separate the values of ${\epsilon }_{\mathrm{opt}}$ in three intervals: for the “No IHHOP” interval the Gaussian-like behavior is dominant in the distribution; for the “With IHHOP” interval the heavy-tail is dominant; for the “Maybe IHHOP” interval both behaviors cannot be clearly separated. The dashed grey line plotted in Fig. 2 represents a Gaussian distribution with a similar shape to the ${\epsilon }_{\mathrm{opt}}$ PDF. In the “No IHHOP” region the ${\epsilon }_{\mathrm{opt}}$ distribution shows great similarity to a Gaussian behavior; in the “With IHHOP” region the Gaussian curve is negligible; as for the “Maybe IHHOP” region the probability for ${\epsilon }_{\mathrm{opt}}$ described by a Gaussian distribution is non-negligible but smaller than the observed probability, making the normal and IHHOP behavior for the oxygen saturation indistinguishable for this range of ${\epsilon }_{\mathrm{opt}}$.

On the right side of Fig. 2, several examples of time series with 5 min segments for the oxygen saturation are plotted, for different values of ${\epsilon }_{\mathrm{opt}}$. The y-axis is omitted to emphasize relative differences between the segments, all have the same scale for oxygen saturation values. Each segment is color-coded to the adequate region in the distribution plot. The segments labeled A, B and C are from the “No IHHOP” range of ${\epsilon }_{\mathrm{opt}}$, and they present an ordered, almost constant value for the oxygen saturation. The C segment is close to the boundary with the “Maybe IHHOP.” Segments D, E, and F are in the “Maybe IHHOP” region. Although they present higher amplitude fluctuations than segments A, B, and C, their behavior is a combination of the normal behavior expected for oxygen saturation and IHHOP. On the other hand, time series G, H, and I present distinct high amplitude fluctuations that are characteristic of the IHHOP phenotype described in this work.

### SAS diagnostic

**Figure 3:**
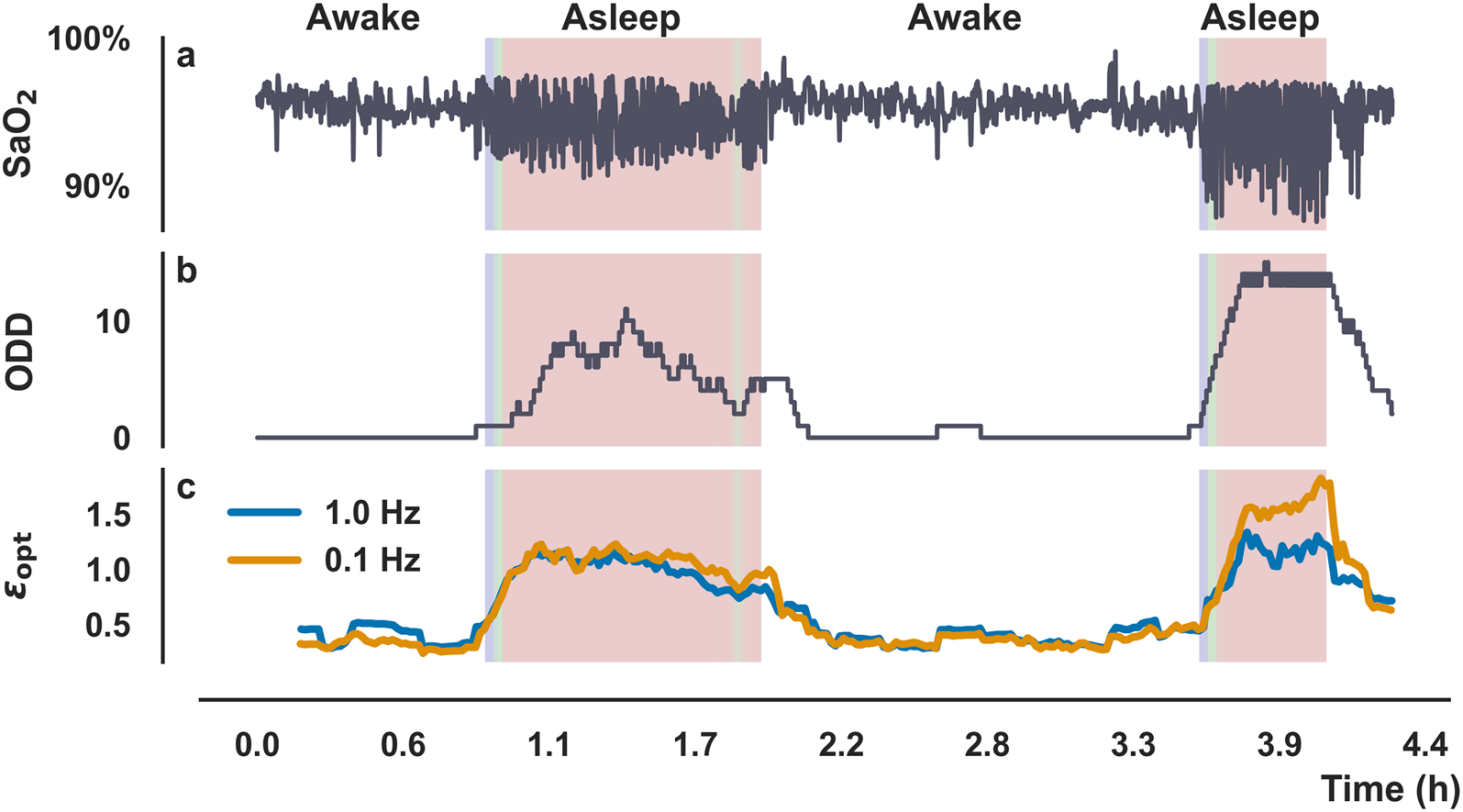
(**a**) Arterial oxygen saturation time series. (**b**) Oxygen Desaturation Density (ODD), ODD was calculated by looking at 10 min windows. (**c**) Rolling window analysis for ${\epsilon }_{opt}$, with a moving rolling window of 10 min long. The blue line corresponds to the value of ${\epsilon }_{opt}$ calculated from a 1 Hz SaO_2_, and the orange line was calculated from SaO_2_ with a 0.1 Hz sampling frequency. The shaded areas correspond to periods in which the patient was reported asleep, color-coded to the intensity of the observed IHHOP.

In Fig. 3a an illustrative SaO_2_ time series is plotted alongside its oxygen desaturation density (ODD) (panel B), and the proposed metrics ${\epsilon }_{\mathrm{opt}}$. The value of ${\epsilon }_{\mathrm{opt}}$ was calculated from SaO_2_ with two distinct sampling frequencies, namely 1 Hz and 0.1 Hz (panel C). In panels B and C, the shaded areas correspond to the periods in which the patient was reported asleep, color-coded to reflect the intensity of the IHHOP, following the same color map from Fig. 2. Regardless of the SaO_2_ sampling frequency, there is a clear correlation between the ODD values and ${\epsilon }_{\mathrm{opt}}$. Since the ODD interpretation is only well defined in the study of sleep apnea, we considered only periods in which the patients were reported asleep to calculate the Pearson correlation ($\rho$) between the two metrics. For a sampling frequency of 1 Hz, $\rho =0.7$; and when the sampling frequency is 0.1 Hz, $\rho =0.76$, which indicates a high correlation between the two metrics, independently of the sampling frequency of the original Oxygen saturation time series. This result is an indication of the robustness of ${\epsilon }_{\mathrm{opt}}$ against different sampling frequencies. It is possible to see in Fig. 3 that the ${\epsilon }_{\mathrm{opt}}$ seems to be more sensitive than the ODD at detecting the occurrence of IHHOP at its onset, which is noticeable by its faster increase around the moment the patient first fell asleep (time ~ 1 h). This higher sensitivity could explain why larger values of correlation to the ODD were not observed.

**Figure 4:**
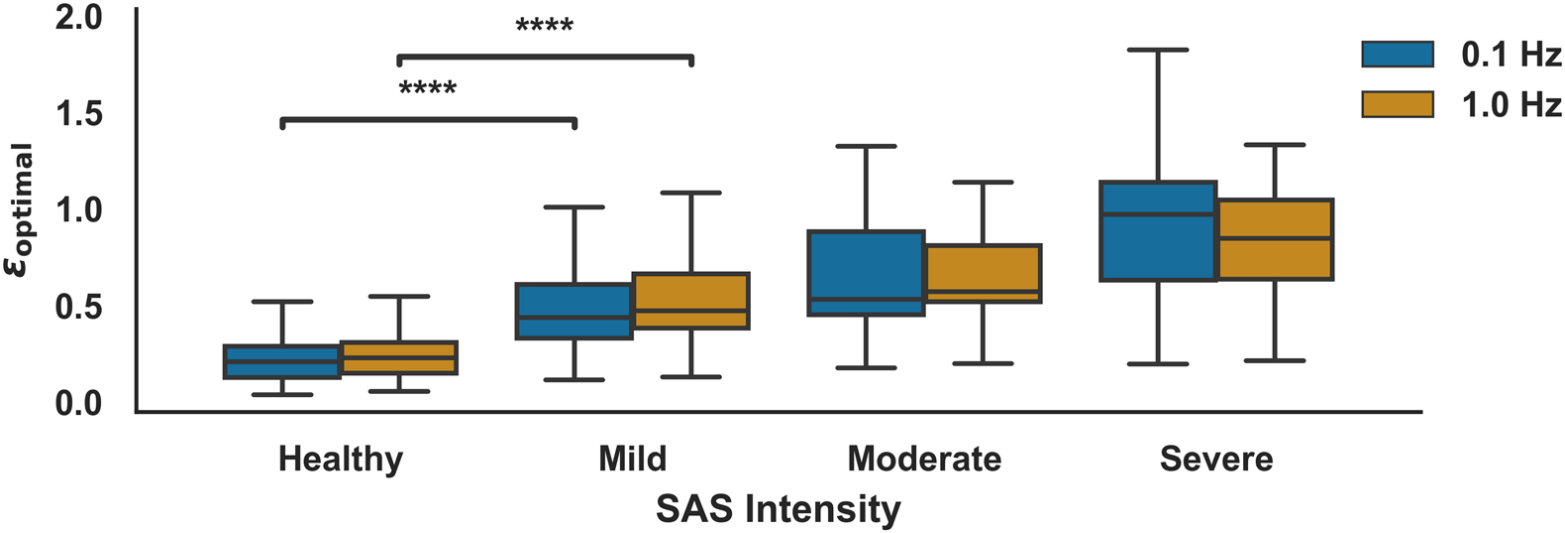
Boxplot with the values of ${\epsilon }_{opt}$ for different SAS intensities calculated by their respective ODD value. ****p-value ≤ 0.0001 for a t-test independent sample.

In Fig. 4 there is plotted a boxplot of the values of ${\epsilon }_{\mathrm{opt}}$ for different SAS intensities. The intensity of SAS was determined following the ASDS classification and using the ODI as a direct surrogate for the AHI^36^. The results are presented for the two different sampling frequencies for the SaO_2_, 0.1 Hz and 1.0 Hz. The calculated value for ${\epsilon }_{\mathrm{opt}}$ increases consistently with the SAS intensity, which suggests it can be used as a diagnostic tool for SAS. Using an independent samples *t*-test between the Healthy and Mild groups, the averages are found to be different with high statistical significance (p-value < 0.0001), regardless of the sampling frequency for the Oxygen Saturation.

Due to the high statistical significance between ${\epsilon }_{\mathrm{opt}}$ and SAS intensity, it can be argued that ${\epsilon }_{\mathrm{opt}}$ can be used as an alternative predictor of SAS-related events in real-time. Considering only reported sleep time, a label was assigned to every 5 min segment based on their ODD value. Using the ASDS classification for OSAHS, based on the AHI value, and using the same values for classification of the ODD, to each time instant was assigned one of the following labels: “Healthy,” “Mild,” “Moderate,” and “Severe.” Then, these labels were translated into two labels “With SAS” and “Without SAS,” so that the data could be subjected to binary classifiers. The organization of the data in the two labels was made according to three different schemes.

- **> Mild:** “Without SAS” was assigned to every segment with the “No SAS” label, and “With SAS” was assigned to every segment with “Mild,” “Moderate” or “Severe” labels.
- **> Moderate** “Without SAS” was assigned to every segment with the “No SAS” and “Mild” labels, and “With SAS” was assigned to every segment with “Moderate” or “Severe” labels.
- **> Severe** “Without SAS” was assigned to every segment with the “No SAS,” “Mild” or “Moderate” labels, and “With SAS” was assigned to every segment with “Severe” label.

**Figure 5:**
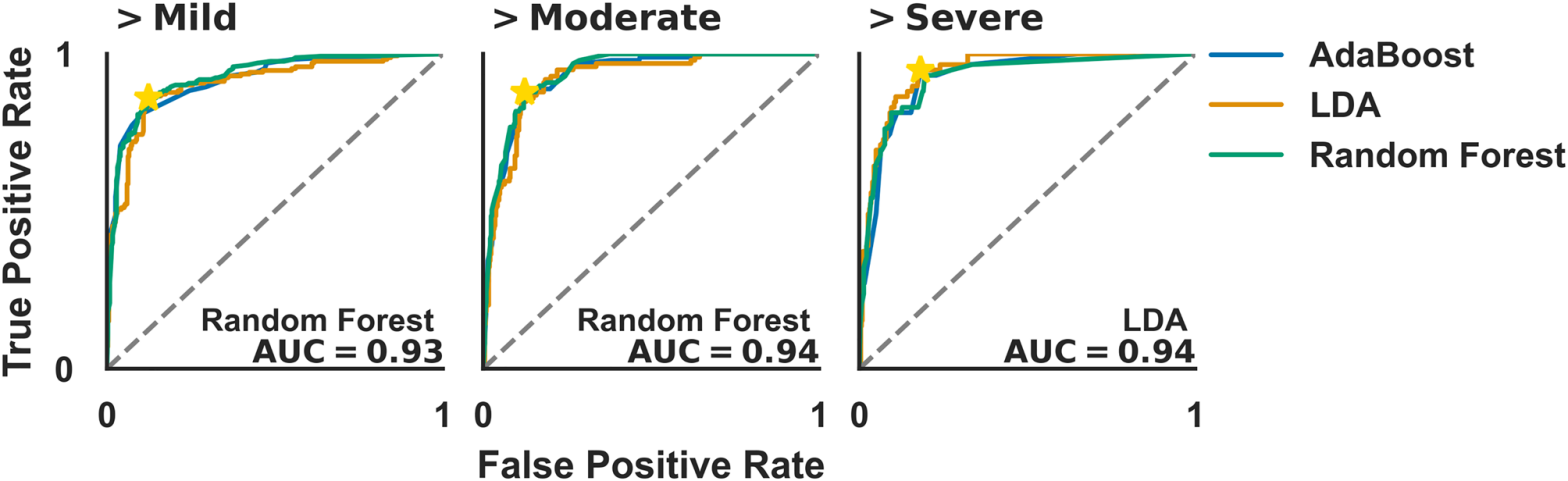
ROC curve for different binary classification algorithms for identifying SAS events based on ${\epsilon }_{opt}$ values. The stars in the figure represent the points chosen for individual analysis of sensitivity, specificity, PPR, and NPR, for the best performing classifier. For each scenario, there is the AUC for the best performing classifier.

Based on this new binary classification, after normalization of the features, the data was subjected to a series of different binary classifiers, namely AdaBoost, Linear Discriminant Analysis (LDA), and Random Forest. There were a total of 1163 5-min segments that were classified as healthy, 244 as mild, 133 as moderate, and 196 as severe. From a recurrence perspective all segments are independent, because they provide a unique comparison of different dynamical regimes within 5 min. A 70%/30% stratified split of training and test set was used to cross-validate the method and prevent overfitting. It is important to note that the classifiers are not being applied to a patient level but rather to an event level, in other words, it is classifying each 5 min segment of the time series according to the presence and intensity of IHHOP. For that reason, both the training and test set may contain segments from the same dialysis session. In Fig. 5 there are the ROC curves for the classifiers, for the three different categorizations of the patients. The area under the curve (AUC) for the best performing classifier is shown on each graph, and they are always larger than 0.92. The performance of the classifiers increases as only the most severe cases are kept in the analysis. Also, all used classifiers yield remarkably high AUC, suggesting that the classification is robust. This result is in accordance with what is observed in the boxplots in Fig. 4. The AUC is a useful metric to determine the overall predictive power of a test over the entire range of values for the test output. However, in a realistic clinical scenario, a cut-off must be chosen that best represents the trade-off between true and false-positive rates (TPR and FPR). For illustrative purposes, we determined the cut-off so as the respective point in the ROC curve (marked in Fig. 5 by a star) is the furthest away from the point with TPR = 0 and FPR = 1. For the best performing classifier, we calculated sensitivity, specificity, positive predictive value (PPV), and negative predictive value (NPV). The results are displayed in Table 2. In all the scenarios, both sensitivity and specificity have values larger than 0.8, confirming the superior performance of the method in correctly identifying the occurrences of SAS (as defined by the ODD). The PPV and NPV, on the other hand, reflect the probability that a positive (negative) test corresponds to a true (false) SAS episode, and they are sensitive to the prevalence of SAS in the studied population. For that reason, the value of PPV decreases for moderate and severe cases since these are much less prevalent in the study. For mild cases, 70% of the predicted SAS episodes correspond to true occurrences.

**Table 2:** Sensitivity, specificity, positive (PPV), and negative predictive values (NPV) for the best performing classifiers.

	1 Hz sampling frequency			0.1 Hz sampling frequency		
	> Mild	> Moderate	> Severe	> Mild	> Moderate	> Severe
Sensitivity	0.88	0.89	0.93	0.84	0.88	0.92
Specificity	0.82	0.85	0.82	0.85	0.83	0.86
PPV	0.71	0.58	0.40	0.73	0.55	0.45
NPV	0.93	0.97	0.99	0.91	0.97	0.99

The results in Fig. 5 were calculated using the time series with a 1 Hz sampling frequency, if the same procedure is implemented for the 0.1 Hz sampling frequency time series the following AUC are calculated: (1) $\mathrm{AUC}=0.902$ with LDA for > Mild; (2) $\mathrm{AUC}=0.921$ with AdaBoost for > Moderate; (3) $\mathrm{AUC}=0.945$ with Random Forest for > Severe. In Table 2 are the results of sensitivity, specificity, PPV, and NPV, for the best-performing classifier. The obtained results follow the same patterns observed for the time series with a 1 Hz sampling frequency. The results are nearly independent of the original time series sampling frequency, suggesting that ${\epsilon }_{\mathrm{opt}}$ could be effectively used in clinical practice to identify the occurrence of IHHOP in SaO_2_ time series.

The apnea severity in the present study was classified for recording instead of for patient, by doing this it is possible to calibrate the method to be sensitive to apnea episodes in real-time. However, the method could also use severity at the patient level by aggregating all the segmental categorization, in which case, our method could also be used as a diagnostic tool to be applied to different patients.

## Conclusions

The use of oximetric data is ubiquitous in medicine as a noninvasive means of collecting vital signs from patients. Using intradialytic SaO_2_ from the Crit-Line device, we observed a distinguishable pattern that can easily differentiate patients experiencing abnormal intradialytic episodes, which are characterized by high-frequency intermittent and/or sawtooth dynamics. These patterns are related to desaturation and arousal in the SaO_2_ measurement. We have shown that ${\epsilon }_{\mathrm{opt}}$ from recurrence analysis can be used as a metric to quantify the occurrence of abnormal behaviors in the time series. It provides a useful diagnostic tool for intradialytic abnormal oxygen saturation time series and provides information about many of the still unknown mechanisms associated with the generation of subtle arousal and desaturation during dialysis. These behaviors might appear qualitatively similar but may show different dynamic features. In many real-world detections of regime and/or behavior transitions, there are several challenges in the identification of precursory patterns before the appearance of conspicuously distinguishable patterns. These challenges may be related to non-uniformity, non-stationarity, nonconstancy, or even the presence of noise in the data samples. The proposed metrics allow us to see precursory patterns prior to the onset of the intermittent and abnormal intradialytic patterns, which could provide additional means of investigating the pathophysiology underlying the IHHOP and their clinical outcomes. Further, the metric appears as an interesting future direction to investigate how such metric may improve the performance of machine learning techniques that classify nonlinear time series.

## Data Availability

Upon request, clinical data will be provided by the authors in compliance with applicable general and local legal and privacy regulations.
